# Frontotemporal lobar degeneration: old knowledge and new insight into the pathogenetic mechanisms of tau mutations

**DOI:** 10.3389/fnagi.2015.00192

**Published:** 2015-10-14

**Authors:** Giacomina Rossi, Fabrizio Tagliavini

**Affiliations:** Division of Neurology V and Neuropathology, Fondazione IRCCS Istituto Neurologico Carlo BestaMilano, Italy

**Keywords:** tau, *MAPT*, mutation, tauopathies, frontotemporal lobar degeneration, pathogenetic mechanisms

## Abstract

Frontotemporal lobar degeneration (FTLD) is a group of heterogeneous neurodegenerative diseases which includes tauopathies. In the central nervous system (CNS) tau is the major microtubule-associated protein (MAP) of neurons, promoting assembly and stabilization of microtubules (MTs) required for morphogenesis and axonal transport. Primary tauopathies are characterized by deposition of abnormal fibrils of tau in neuronal and glial cells, leading to neuronal death, brain atrophy and eventually dementia. In genetic tauopathies mutations of tau gene impair the ability of tau to bind to MTs, alter the normal ratio among tau isoforms and favor fibril formation. Recently, additional functions have been ascribed to tau and different pathogenetic mechanisms are then emerging. In fact, a role of tau in DNA protection and genome stability has been reported and chromosome aberrations have been found associated with tau mutations. Furthermore, newly structurally and functionally characterized mutations have suggested novel pathological features, such as a tendency to form oligomeric rather than fibrillar aggregates. Tau mutations affecting axonal transport and plasma membrane interaction have also been described. In this article, we will review the pathogenetic mechanisms underlying tau mutations, focusing in particular on the less common aspects, so far poorly investigated.

## Introduction

Alzheimer (1907) described the existence in brain tissue of intracellular neurofibrillary tangles associated with extracellular plaques and only a few years later the presence of intracellular inclusions alone in a different case of dementia (Alzheimer, 1911).

It was not until the 1980s that the protein constituting these deposits was identified as the microtubule-associated tau protein (Goedert et al., 1988; Wischik et al., 1988). Tau protein had been previously identified in 1975 as a new microtubule-associated protein (MAP) with a relatively low molecular weight with respect to high molecular weight MAP-1 and MAP-2 (Weingarten et al., 1975).

Primary tauopathies belong to the frontotemporal lobar degeneration (FTLD) group of degenerative diseases. FTLD are heterogeneous from a clinical, pathological and genetic point of view. Clinically, they are characterized by behavior, executive and language impairment, giving rise to behavioral variant of frontotemporal dementia (bvFTD), semantic dementia (SD) and progressive non-fluent aphasia (PNFA), often associated with parkinsonism or motor neuron disease (MND). Pathologically, deposits of different misfolded proteins are found in brain tissue: tau, transactive response DNA binding protein 43, fused-in-sarcoma, and dipeptide-repeat proteins. The main mendelian genes so far linked to FTLD include genes coding for microtubule-associated protein tau (*MAPT*) and progranulin (*GRN*), and Chromosome 9 Open Reading Frame 72 (*C9ORF72*) gene, with other genes being more rarely associated (Rademakers et al., 2012; Mori et al., 2013).

Primary tauopathies are defined by the presence in neuronal and glial cells of deposits of misfolded, insoluble and hyperphosphorylated tau proteins. Clinically, they fall into the spectrum of FTLD, presenting with bvFTD, SD and PNFA; in addition they also can present with progressive supranuclear palsy (PSP) and corticobasal syndrome (CBS). Tauopathies can be sporadic or familiar, genetically determined by mutations in *MAPT*. As a MAP, the role of tau is to promote microtubule (MT) assembly and stabilization and regulate MT dynamics (Goode and Feinstein, 1994; Gustke et al., 1994; Trinczek et al., 1995). A single *MAPT* gene gives rise to six tau isoforms by alternative RNA splicing of exons 2, 3 and 10. The binding region to MT (microtubule-binding domain, MBD) is in the C-terminal half of tau and is constituted by either 3 repeats (3R tau) or, if exon 10 is included, 4 repeats (4R tau) of 31–32 aminoacids. Alternative splicing of tau is developmentally regulated and in the adult human brain all the six isoforms are expressed, with 3R tau and 4R tau represented at about the same level (4R/3R ratio ≅ 1; Goedert et al., 1989a,b). Since 4R and 3R tau isoforms assume complex and distinct MT binding structures (Goode et al., 2000) and regulate dynamic instability of MT in different ways (Levy et al., 2005), with 4R tau isoforms having a higher ability to promote MT polymerization (Goedert and Jakes, 1990), any imbalance of the 4R/3R ratio is supposed to cause MT misregulation and a tendency of the isoforms in excess to produce fibrillar aggregation.

While in Alzheimer’s disease (AD) tau fibrils are made of all six isoforms (Goedert et al., 1992), in primary tauopathies some isoforms predominate, depending on the neuropathological phenotype. In Pick’s disease (PiD) 3R tau isoforms are present, while in PSP and corticobasal degeneration (CBD) 4R tau isoforms are found. In genetic tauopathies, 4R, 3R or a combinations of 4R and 3R tau isoforms are present (Dickson et al., 2011).

Tau is a highly phosphorylated protein: there are 79 putative phosphorylation sites on the protein and at least 30 have been demonstrated to be actually phosphorylated. The phosphorylation state is the main system which regulates tau binding to MT: non phosphorylated sites lead to a stronger binding, whereas phosphorylation decreases the binding, making MT more unstable (Buée et al., 2000). Hyperphosphorylation characterizes abnormal tau present in all the taupathies (Buée et al., 2000; Spires-Jones et al., 2009).

Although tau is abundantly expressed in central nervous system and is the major MAP of neurons, it is also present in several non-neural tissues, such as fibroblasts and lymphocytes (Ingelson et al., 1996; Thurston et al., 1996; Rossi et al., 2008a).

In 1990s, linkage analysis in families affected by frontotemporal dementia with parkinsonism (FTDP) and pathologically characterized by tau deposits in neuronal and glial cells indicated that the candidate gene lied at 17q21–22, where *MAPT* is located, and in 1998 sequencing analysis finally revealed pathological mutations of *MAPT* (FTDP linked to chromosome 17-tau, FTDP-17T). Some missense mutations were recognized as causative based on the highly conserved site, the absence in control subjects and the segregation with the disease in the FTDP-17T families: G272V, P301L and R406W mutations (Hutton et al., 1998). A different kind of mutations was also found in other families: base pair substitutions in the 5′ splice site of exon 10 (intron 10). They segregated with the disease and were not present in control subjects (Hutton et al., 1998; Spillantini et al., 1998). Several further missense and splice-site mutations, as well as in-frame small deletions, were later found. All the mutations are transmitted with a dominant pattern of inheritance, with rare exceptions. Penetrance is usually complete, with a few exceptions (van Herpen et al., 2003; Rossi et al., 2008b). Afterwards, different kinds of mutations, such as gross deletions or insertions, regulatory and risk factors have been found. Complete mutation databases to refer to are the Human Gene Mutation Database (Stenson et al., 2014) and the AD&FTD Mutation Database (Cruts et al., 2012).

**Figure 1:**
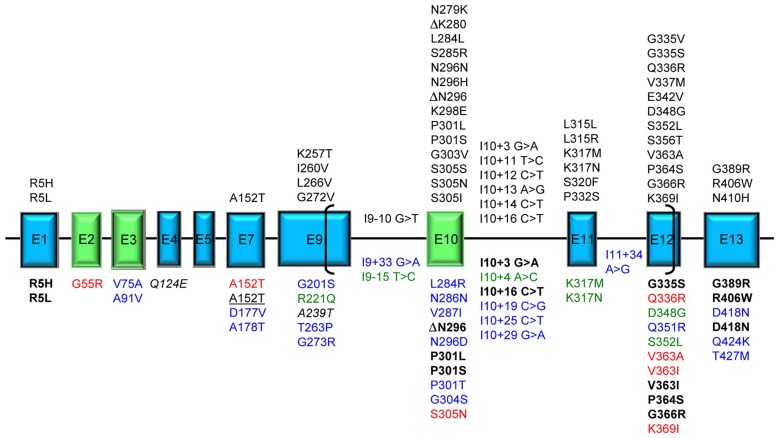
**Tau mutations.** Exons expressed in central nervous system (CNS), producing the longest 441 aa 2N4R tau isoform, are represented as boxes roughly proportional to their relative sizes; introns are represented as horizontal lines and are not proportional to their sizes. Exons subjected to alternative splicing are in green. Brackets include the region of microtubule binding domain (MBD). Above the exon boxes are reported the mutations pathogenic by genetic or neuropathological or functional evidence [reducing microtubule (MT) polymerization or increasing fibril formation]. Below the exon boxes, the mutations are classified by different colors: bold black: displaying unusual pathogenetic mechanisms; red: affecting MT polymerization or fibril formation in uncommon ways; blue: uncharacterized found in frontotemporal lobar degeneration (FTLD) or Alzheimer’s disease (AD) patients; green: atypical by clinical, neuropathological or transmission pattern features; italic: risk factors for tauopathy when associated with mutations in other genes; underlined: risk factors for tauopathy (FTLD or AD). A single mutation can have different features and be reported more than once in the figure.

We present here an overview of all tau mutations so far described and the underlying pathological mechanisms. Figure 1 represents an overall view of all tau mutations whose pathogenicity is definite/probable by means of genetic, neuropathological or functional data; mutations with peculiar features, mutations uncharacterized but present only in affected subjects and mutations considered as risk factors are also indicated.

## Mutations Affecting MT Polymerization and/or Fibrillar Aggregation

### Mutations Within or near the MBD and in C-Terminal Domain

#### Missense Mutations

**Figure 2:**
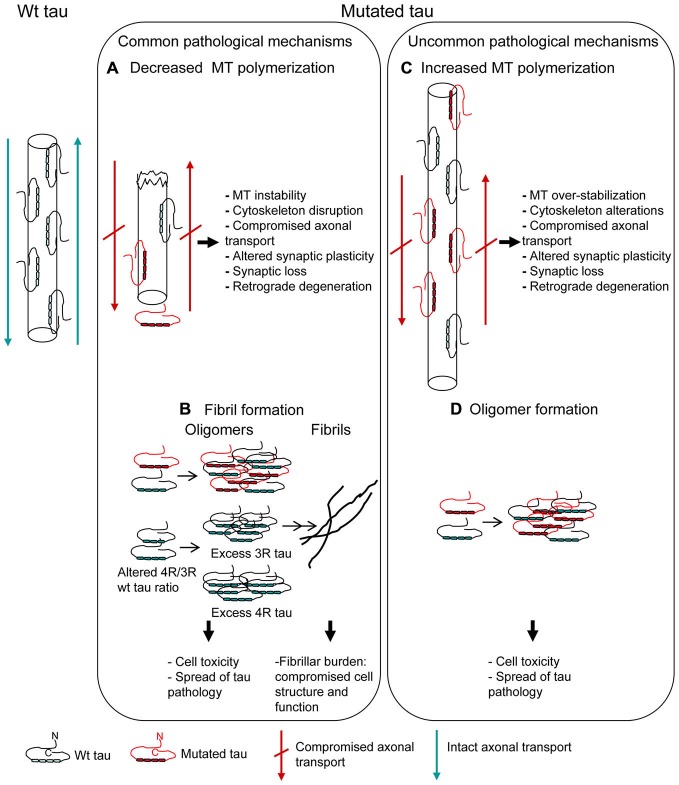
**Mutations affecting MT polymerization and/or fibrillar aggregation.** Pathogenetic mechanisms underlying tau mutations are depicted. Most of tau mutations reduce the protein ability to promote MT polymerization **(A)** and/or increase the propensity to aggregate into fibrils **(B)** (“Common pathological mechanisms”). However, a few tau mutations act in the opposite way, increasing MT polymerization **(C)** and/or decreasing the propensity to aggregate **(D)** (“Uncommon pathological mechanisms”). Alterations of MT dynamics, both as destabilization or over-stabilization, may anyway lead to cell degeneration. Oligomer toxicity (interaction with cell membrane, calcium uptake, mithocondrial dysfunction) is now widely confirmed; fibrillar aggregation, even if it might be considered as an attempt by the cell to sequester and neutralize oligomer toxicity, is however a structural and functional burden to cell.

The MBD (exons 9–12; aminoacid 244–368; Mukrasch et al., 2005) is the region essential for the primary function of tau, that is MT binding, polymerization and dynamics regulation. By means of *in vitro* affinity or polymerization assays of recombinant tau protein with monomeric tubulin, most of missense mutations localized in MBD have been demonstrated to confer a reduced ability to interact with tubulin, slowing and decreasing MT formation (Hasegawa et al., 1998; Hong et al., 1998; Figure 2A). This “loss of function” pathological mechanism can lead to cytoskeleton disruption, affecting physiological cell functions (Iqbal et al., 2009).

G389R and N410H mutations have a reduced ability to promote MT polymerization (Murrell et al., 1999; Pickering-Brown et al., 2000; Kouri et al., 2014) although localized outside the repeats of MBD, in the C-terminal domain (exon 13). However, this region is highly conserved across species, is close to MDB and, most importantly, intramolecular interactions among different tau regions, including C-terminal and N-terminal domains and MBD, have been reported (Jeganathan et al., 2006; Mukrasch et al., 2009). Thus, mutations in C-terminal domain may influence MT formation.

MBD contains two short sequences responsible for the acquisition of β-structure necessary for tau aggregation into pathological fibrils (Mukrasch et al., 2005). Thus, several missense mutations, depending on their position, also increase the propensity of tau to aggregate into fibrils (Barghorn et al., 2000; von Bergen et al., 2001) (Figure 2B), although some pathogenic mutations did not show this effect (Neumann et al., 2001; Yoshida et al., 2002; Rossi et al., 2012). Fibrillar burden represents a “gain of function” pathological mechanism, with aggregated tau being detrimental to cell function (Spillantini and Goedert, 2013).

Some mutations (V337M, G272V and P301L) have been shown to make tau a more favorable substrate for phosphorylation by brain protein kinases (Alonso et al., 2004). This in turn promotes the reduced binding to MT and the self-aggregation (Alonso et al., 2010). In fact V337M, G272V and P301L have both a reduced binding to MT an increased propensity to aggregation (Hasegawa et al., 1998; Barghorn et al., 2000; Chang et al., 2008).

R406W mutation, in the C-terminal domain, showed contradictory features: it appeared to have a reduced ability to promote MT binding in *in vitro* assays (Hasegawa et al., 1998; Hong et al., 1998) but non in a microinjected-cell system (Bunker et al., 2006). It was reported to favor tau phosphorylation in an *in vitro* system (Alonso et al., 2004) and to decrease it in cellular systems (Vogelsberg-Ragaglia et al., 2000). It has an ability to aggregation similar to wt tau (Chang et al., 2008). Anyway, this mutation showed peculiar pathological mechanisms (see below) that are perhaps the most relevant to neurodegeneration.

#### Intron 10 Splicing Mutations

These mutations are located at the 5′ splice site of exon 10, that is within the first bases of intron 10. A RNA stem-loop structure possibly involved in regulating the alternative splicing of exon10 was predicted (Hutton et al., 1998) and afterwards the three-dimensional structure of this region was determined (Varani et al., 1999), showing an upper and lower stem with an apical loop. This structure is involved in regulating alternative splicing of exon 10 presumably by hampering the U1 snRNP binding to the site. Blocking U1 snRNP binding would result in failure to include exon 10 and lead to skipping of this exon (Hutton et al., 1998). The splicing mutations, located in the upper stem (Spillantini and Goedert, 2000), destabilize the structure, causing a more frequent usage of the 5′ splice site and an increased inclusion of exon 10 in the mRNA, with an overproduction of 4R tau transcripts and the imbalance of 4R/3R tau isoforms ratio. The excess of 4R tau isoforms leads to their accumulation in the cytoplasm followed by formation of fibrillar deposits (Buée et al., 2000).

#### Silent Mutations and Missense Mutations Affecting Exon 10 Splicing

These mutations can be located either in the stem-loop structure (S305S, S305N, S305I), producing the same effect as the intron 10 splicing mutations (Hasegawa et al., 1999; Stanford et al., 2000; Kovacs et al., 2008), or within regulatory elements (enhancers or silencers) in exon 10 (L284L, N286N, N296N; N279K, N296H) affecting exon 10 splicing in a cys-acting manner.

N279K and L284L mutations strenghten two different regions of an enhancer, both resulting in an increased inclusion of exon 10 that is an increase of 4R tau transcripts (D’Souza et al., 1999; Hasegawa et al., 1999; D’Souza and Schellenberg, 2002). N296N and N296H mutations are supposed to disrupt a silencer (D’Souza and Schellenberg, 2002) or, alternatively, to create a new enhancer (Grover et al., 2002), with the same result of increasing 4R tau transcripts. N286N mutation was supposed to be pathological in the same way (Rohrer et al., 2009).

Some of the missense mutations were also reported to affect protein function. N279K mutation was described to reduce MT polymerization by some authors (Barghorn et al., 2000) but not by others (D’Souza et al., 1999; Hasegawa et al., 1999) and to increase the self-aggregation into fibrils (Barghorn et al., 2000). N296H mutation was described to reduce MT polymerization (Grover et al., 2002); the increase of fibril formation is controversial (Grover et al., 2002; Yoshida et al., 2002).

Recently, N410H mutation in exon 13 was reported to increase the 4R/3R tau mRNA ratio. The increase of 4R tau mRNA had also been previously reported for the E342V tau mutation in exon 12 (Lippa et al., 2000). This raises the question of how mutations localized outside exon 10/intron 10 can alter the exon 10 splicing. Unknown regulatory elements (enhancers/silencers) may possibly exist in different regions of *MAPT* gene.

#### Microdeletions

Two in-frame microdeletions involving each a single codon have been described: ΔN296 e ΔK280. ΔN296 mutation was consistently reported to reduce MT polymerization, while increasing of fibril formation and effect on exon 10 splicing were controversial (Grover et al., 2002; Yoshida et al., 2002).

Thus, it has to be taken into account the fact that some mutations (N279K, N296H and ΔN296) can lead to an overproduction of tau 4R transcripts that however, because of the presence of the mutation, has a lower ability to promote MT polimerization.

ΔK280 is a very peculiar and puzzling mutation. This deletion (the deletion is of an AAG that actually can be either at codon 280 or 281, thus the mutation may be named ΔK280 or ΔK281) in exon 10 was first described by Rizzu et al. (1999) in a patient affected by FTD. They also showed that recombinant mutated protein had a dramatically reduced ability to promote MT assembly. The relevance of this codon to MT affinity and dynamic instability had already been reported in *in vitro* studies some years before (Goode and Feinstein, 1994; Trinczek et al., 1995).

Furthermore, tau carrying ΔK280 mutation is highly fibrillogenic and has been used as a model of tau aggregation, even in absence of aggregation inducers such as heparin (Barghorn et al., 2000; von Bergen et al., 2001; Larini et al., 2013).

However, in addition, ΔK280 mutation nearly abolished the exon 10 splicing *in vitro*, leading to an overproduction of 3R tau isoforms (D’Souza et al., 1999), presumably disrupting an enhancer element (D’Souza and Schellenberg, 2002). The preponderance of 3R tau was confirmed by neuropathological examination (van Swieten et al., 2007; Momeni et al., 2009). Since all the others pathogenic tau mutations affecting exon 10 splicing increase 4R tau, ΔK280 is a rather peculiar mutation. It can only be present in 4R tau, being in exon 10. Thus, the *in vitro* described pathological effects on MT and tau aggregation can only be accomplished by mutated 4R tau. However *in vivo* mostly 3R tau is present, raising some doubts about the pathogenicity of this mutation. Possible explanations of its pathogenicity may be that: (i) even the very small amount of highly fibrillogenic ΔK280 4R tau is sufficient to begin tau deposition and sequester wt 3R or 4R tau, altering MT dynamics and (ii) overproduction of 3R tau leads to accumulation of this isoform into fibrillar deposits.

#### Mutations Atypical by Clinical, Neuropathological or Transmission Pattern Features

A family affected by lower MND and respiratory failure was described (Di Fonzo et al., 2014) in which D348G mutation (exon 12) segregated with the disease with a dominant pattern.

Neuropathological examination disclosed pathological tau deposits in hippocampus and motor neurons. D348G mutation seemed not to alter the ability of tau to bind to MT, at variance with the majority of the other mutations localized in the MBD. However this result is based on the evaluation of relative amounts of acetylated/tyrosinated tubulin and on a MT-binding assay in transfected cells which may not be comparable to the *in vitro* MT polymerization assay used to study the other mutations. The latter assay may in fact be more sensitive and reveal minor differences (Hasegawa et al., 1998; Hong et al., 1998).

A mutation very near to D348G was reported, S352L, interestingly displaying a similar clinical phenotype of respiratory failure, despite the different age of onset, and an atypical neuropathological phenotype (Nicholl et al., 2003). Mutated protein showed a reduced ability to promote MT polymerization and an increased propensity to aggregate. This mutation was transmitted with a recessive pattern of inheritance, the only example for *MAPT* and one of the very few cases in neurodegenerative diseases.

Another unique case in *MAPT* genetics was the finding of a family with compound heterozygous mutations I10 + 4 A > C; I9 – 15 T > C (Anfossi et al., 2011). The heterozygous condition was associated with normal phenotype and did not alter the R4/R3 tau ratio, while the compound heterozygous state was linked to FTD and increased R3 tau isoforms both *in vitro* and at neuropathological examination, where a Pick-like picture was shown.

I10 + 4 A > C mutation may be in a position not so critical to strongly influence exon 10 splicing; similarly, although the I9 – 10 G > T mutation was reported, segregating with FTD and increasing 4R tau isoforms (Malkani et al., 2006), I9 – 15 T > C mutation may be in a less critical position and have scarce effect. Only the contemporary presence of the two mutations surrounding exon 10 could significantly alter its splicing, supposing trans-acting effects on splicing machinery.

The increase of 3R tau isoforms in association with tau mutations is not frequent: to date, only ΔK280, I10 + 19 C > G (Stanford et al., 2003) and this compound heterozygous mutations showed this effect. Most of splicing affecting mutations, in contrast, produce an increase of 4R tau isoforms. However, the imbalance between 4R and 3R tau isoforms appears to be pathological in any case.

K317M is the only tau mutation so far reported causing FTLD and MND (Zarranz et al., 2005). In fact, FTLD-MND is mostly associated with mutations in *C9ORF72* gene (Rohrer and Warren, 2011). K317N is the first mutation to date reported associated with the recently defined pathological entity globular glial tauopathy (GGT; Tacik et al., 2015). After reviewing the neuropathological study, the Authors suggest that K317M also may be classified as GGT.

R221Q mutation was identified in a sporadic patient diagnosed with Dementia with Lewy bodies (DLB): this is the first case of probable DLB carrying a *MAPT* mutation (Meeus et al., 2012). However, the mutation is outside the MBD, and no functional studies were performed, thus its pathogenetic character is so far not proven.

### Missense Mutations in the N-Terminal Domain

R5L and R5H mutations (exon 1) were detected in patients affected by PSP and late-onset frontotempral dementia, respectively (Hayashi et al., 2002; Poorkaj et al., 2002). Both mutations affected MT assembly and R5H, in addition, showed an increased propensity to aggregation. G55R is the first mutation found in the alternatively spliced exon 2. Similarly to R5L and R5H mutations, it affects MT assembly, although with a unusual mechanism (see below).

How a mutation in N-terminal domain may affect the functionality and aggregation properties of the MBD can be speculated. While it has been showed that flanking regions of MBD act as “targeting” domains and the 3 or 4 repeats act as “catalytic” domains for MT polymerization (Trinczek et al., 1995), the N-terminal domain did not appear to contribute. However, as already discussed for C-terminal mutations, intramolecular interactions among C-terminal and N-terminal domains and MBD have been reported (Jeganathan et al., 2006; Mukrasch et al., 2009). Thus, these N-terminal mutations may affect MBD properties. Additional hypotheses about the pathological effects of R5L and R5H mutations have been advanced after the interaction of tau with the dynactin complex was demonstrated (Magnani et al., 2007; see below).

### Mutations Affecting MT Polymerization and/or Fibrillar Aggregation in an Uncommon Way

Usually, tau mutations affecting the interaction with MT cause a reduced ability to promote MT polymerization and the mutations affecting tau self-aggregation lead to fibril formation. However a few mutations showed different abnormal features (Figures 2C,D).

V363I mutation has been associated with several FTLD clinical phenotypes and also with an uncommon phenotype such as posterior cortical atrophy (Rossi et al., 2014a). Neuropathological examination is unfortunately missing, but *in vitro* functional and structural analysis revealed peculiar and very likely pathological features. V363I mutation showed a greater ability to promote MT polymerization, consisting in both a higher polymerization rate and the formation of longer tubulin polymers (Rossi et al., 2014a; Figure 2C). The increased ability of tau to induce MT polymerization had been previously reported for Q336R and S305N tau mutations (Pickering-Brown et al., 2004), but the effect was small or unclear and not further investigated. G55R mutation was recently described to increase MT polymerization, although MT length was smaller than for wt tau (Iyer et al., 2013). Increased MT polymerization can be considered as a “gain of function” pathological mechanism altering the tightly regulated MT dynamics, which is necessary for physiological neuronal functions such as axonal transport and synapse plasticity. In fact, excessive MT polymerization, although due to excess of wt tau rather than to mutated tau, produced axonal dilations and motor impairment in a transgenic mouse model (Spittaels et al., 1999), which could be rescued by reducing the ability of tau to bind to and promote MT polymerization (Spittaels et al., 2000).

V363I mutation showed a dramatically reduced tendency to fibrillogenesis, producing instead oligomers that persist over long incubation times without any fibrils production; similar properties were shown by V363A mutation (Rossi et al., 2014a; Figure 2D). Other two tau mutations previously showed a similar behavior: K369I mutation and the risk factor A152T, which however caused the formation of shorter than wt fibrils and not merely oligomers (Neumann et al., 2001; Coppola et al., 2012). Toxicity of tau oligomers rather than, or in addition to, tau fibrils is now emergin. Both extracellular (Gómez-Ramos et al., 2008; Lasagna-Reeves et al., 2010) and intracellular (Iliev et al., 2006) tau oligomers were toxic to neuroblastoma cells. Tau oligomers have been isolated from brains affected with AD (Maeda et al., 2006) and a correlation between the presence of tau oligomers and memory loss was described in animal models (Berger et al., 2007). In addition, tau oligomers are considered as responsible for the spread of tau pathology (Gerson and Kayed, 2013). Thus, tau oligomer formation and persistence may be considered a pathological feature of mutated tau protein.

## Genetic Risk Factors for Tauopathies

Genetic risk factors are sequence variations that can be present both in healthy controls and affected subjects but with significantly different proportions, reflecting their character predisposing to disease. They can be localized in coding regions or in regulatory regions, affecting different features of a gene.

### H1/H2 MAPT Haplotypes

After the first identification of the significant allelic association of a *MAPT* polymorphism with PSP (Conrad et al., 1997), the allelic association was subsequently extended to a series of polymorphisms covering the entire *MAPT* coding region, allowing the definition of the haplotypes H1/H2 (Baker et al., 1999). A more extended haplotype spanning promoter, intronic and coding regions of *MAPT* was then described and H1 haplotype was found associated with PSP and CBD (Pittman et al., 2005; Cruchaga et al., 2009). Chromosome 17q21, where *MAPT* is located, is characterized by a peculiar genomic architecture. In fact, the presence of low-copy repeats is at the basis of the 900 kb inversion that resulted in the formation of common H1 and H2 haplotypes (Cruts et al., 2005; Stefansson et al., 2005).

**Figure 3:**
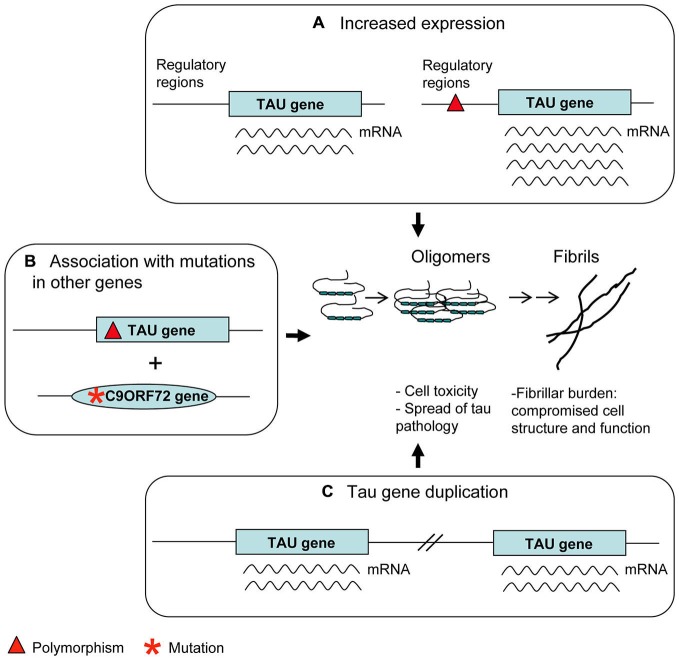
**Genetic risk factors.** Polymorphisms in regulatory regions **(A)** as well as Tau gene duplication **(C)** may account for an increased tau expression. This is considered a disease risk factor because of the possible tendency of tau molecules in excess to aggregate into oligomers and fibrils. Some apparently benign tau polymorphisms **(B)** can produce an overt tau pathology only in association with mutations present in other genes relevant to neurodegenerative diseases (*C9ORF72* is an example).

Some of the haplotype polymorphisms were characterized for their possible functional significance in the context of the disease (Figure 3A).

– H1c sub-haplotype apparently gives rise to an increased expression of tau, mostly 4R tau transcripts (Myers et al., 2007). Notably, H1c haplotype was associated with increased risk for PSP (Pittman et al., 2005), in which 4R tau deposits are present, although these results were not replicated (Cruchaga et al., 2009).
– The 5′-upstream variant rs1880753, localized at 160 Kb from transcription start site of *MAPT*, is associated with PSP and CBD; this variant is in a conserved sequence across mammals which is a potential binding site for YY1 and BNCF/BNC transcription factors (Cruchaga et al., 2009). Similarly, the −221 C > G and −44 A > G polymorphisms in the promoter region, associated with PSP, could affect binding sites for transcription factors (de Silva et al., 2001).
– The +347 G > C polymorphism in the promoter up-regulates *MAPT* expression in an *in vitro* assay (Sun and Jia, 2009).

Deregulated and in particular increased expression of tau may lead to the risk of a pathological accumulation and deposition of the protein in its fibrillar form, as confirmed by several studies on animal models of tauopathies in which tau is over-expressed (Götz et al., 2007).

### Missense Mutations

Nearly all missense mutations inside or near the MBD have been demonstrated to be pathogenic and benign polymorphisms in this region of *MAPT* are so far absent, with the exception of V300I (exon 10). This is the only polymorphism described in MBD (Guerreiro et al., 2010); however it can not be excluded that the subject in whom it was detected could be a presymptomatic case, as no details about this individual are available. All the remaining missense mutations in this region must be considered risk factors or mutations not well characterized.

A239T mutation (exon 9) is a very peculiar case. It was first described in a patient affected by FTD with tau-negative neuropathology (Pickering-Brown et al., 2002). The finding in the same patient of a *null* mutation in *GRN* suggested A239T to be a very rare benign variant (Pickering-Brown et al., 2006). However, the possible pathological role of A239T was raised again by the interesting finding that another patient carrying the *C9ORF72* expansion and the A239T had, at neuropathological examination, a tauopathy overhanging p62 and TDP-43 pathology (King et al., 2013). Thus, A239T mutation may be a risk factor for tauopathy appearing preferably in association with other pathogenetic mutations (Figure 3B). Similar considerations can be made about the Q124E mutation (exon 4), associated with a *LRRK2* mutation (Ling et al., 2013).

A152T mutation (exon 7) has been demonstrated to be a risk factor for tauopathies, both FTLD and AD, based on neuropathological, association and functional studies (Kovacs et al., 2011; Coppola et al., 2012; Jin et al., 2012; Kara et al., 2012). Although this mutation is far from the MBD, it decreased the binding of tau to MT and their polymerization and showed a tendency to form oligomeric aggregates instead of fibrils, raising the hypothesis that other not well characterized regions of tau may be involved in regulating protein function.

Several missense mutations have been exclusively detected in patients affected by FTLD or AD and not in control subjects; some of them are at same codons where different pathogenic mutations had been previously reported (L284R, P301T, N296D). However segregation, neuropathological or functional studies were not performed, so that their pathogenicity was not proven: V75A, A91V (one case of Parkinson disease), D177V, A178T, G201S, T263P, G273R, L284R, N286N, V287I, N296D, P301T, G304S, Q351R, D418N (one case with ataxia and cognitive impairment), Q424K, T427M (Stenson et al., 2014; Cruts et al., 2012; Jin et al., 2012; Kim et al., 2014). Clinical phenotypes other than FTLD, in particular AD phenotype, were sometimes associated with tau mutations. However it was recognized that this was most likely due to misdiagnosis (Cruchaga et al., 2012).

### Intronic Mutations

A few intronic mutations were found whose pathogenicity is uncertain.

I10 + 29 G > A segregated with the FTLD phenotype in one family but no tauopathy was present in brain tissue. This mutation had been detected at low frequency in control subjects and later a *null* GRN gene mutation was found in this family, suggesting I10 + 29 G > A to be a benign polymorphism (Stanford et al., 2003; Pickering-Brown et al., 2006). I10 + 19 C > G was found in two cases (Stanford et al., 2003; Rohrer et al., 2009) and I9 + 33 G > A in one case (Rizzu et al., 1999) of FTLD; however, they have not been definitely characterized. I10 + 25 C > T was detected both in affected and control subjects (Roks et al., 1999).

I11 + 34 A > G was reported to increase risk factor for AD when associated with ApoE 4 allele (Bullido et al., 2000).

## Gross Deletions or Duplications

The only gross deletion involving a part of *MAPT* and giving rise to a FTLD phenotype was reported by Rovelet-Lecrux et al. (2009). The deletion spanned exons 6–9 and produced a truncated tau devoid of its first MBD. This tau showed pathological features such as a decreased binding to MT and the ability to sequester other MAPs, both impairing MT dynamics.

All the other deletions so far described are in fact deletions spanning large chromosome regions including *MAPT* and adjacent genes, that caused the so-called microdeletion syndromes, mostly characterized by mental retardation, developmental delay and dysmorphisms (Koolen et al., 2006; Sharp et al., 2006; Varela et al., 2006; Dubourg et al., 2011; Asadollahi et al., 2014). In these cases the contributions of the single genes, such as *MAPT*, to the syndrome are not clearly understood.

Duplication cases are more interesting (Figure 3C). In the first case, following a screening of FTLD patients, a duplication was found, spanning four genes, including *MAPT* (Rovelet-Lecrux et al., 2010). In the second case, following a screening of familial AD cases, a duplication of the solely *MAPT* was discovered (Hooli et al., 2014). Unfortunately, neuropathological examination was not available in either cases; however the phenotype of dementia (FTLD or AD) seems to indicate a detrimental role of tau overexpression, accordingly to data from animal models (Spittaels et al., 1999; Andorfer et al., 2003). More recently, a very young case carrying a triplication of *MAPT* gene together with other genes was described to have moderate intellectual disability (Gregor et al., 2012).

These rearrangements are thought to be fostered by the genomic structure itself of this region containing the low-copy repeats and the inversion polymorphism (Cruts et al., 2005, 2012; Sharp et al., 2006).

## Unusual Pathogenetic Mechanisms Underlying Tau Mutations

In addition to the role of MAP, other functions are ascribed to tau, which is presently considered a multifunctional protein (Morris et al., 2011) and a number of tau binding partners have been reported, including proteins, nucleic acids and organelles (Mandelkow and Mandelkow, 2012). Here, we only describe the functions that have been demonstrated to be compromised by a tau mutation. Mutations altering these various functions may sustain pathogenetic mechanisms different from the usual ones.

### Tau can Regulate Axonal Transport

Tau can affect the binding of both dynein and kinesin motor proteins to MT, based on the distribution of tau along MT and the sensitivity of motor proteins to presence of tau (Dixit et al., 2008).

**Figure 4:**
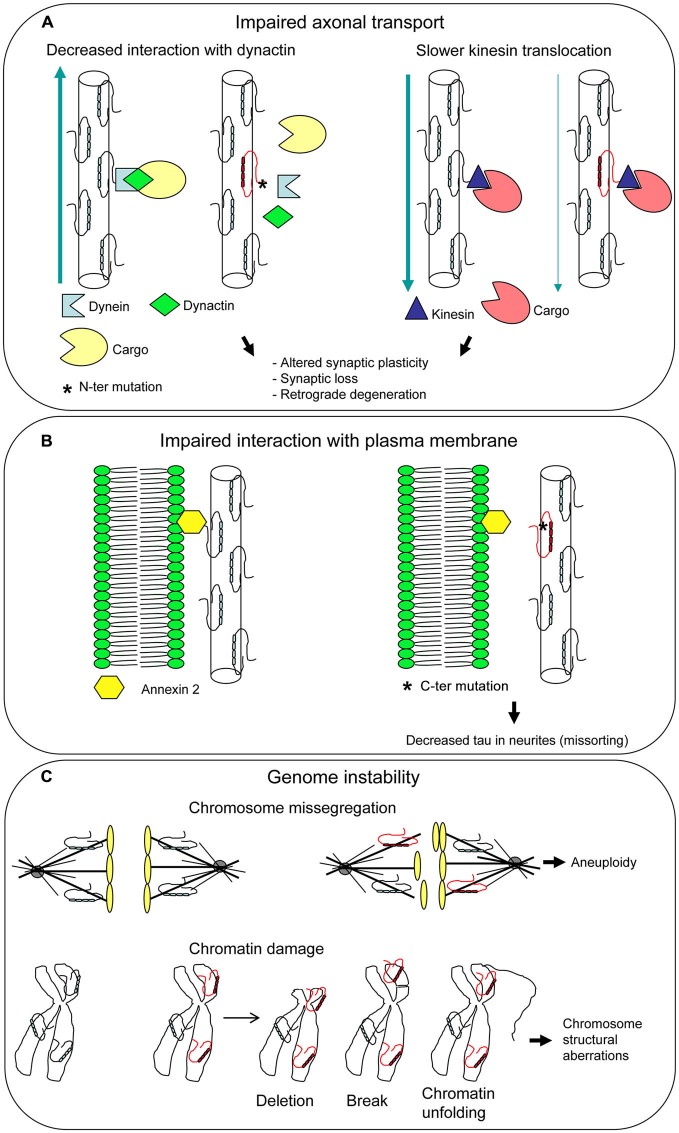
**Unusual pathogenetic mechanisms underlying tau mutations.** Additional functions have been attributed to tau besides its role as a microtubule-associated protein (MAP). Mutations can affect all tau functions, disclosing new pathogenetic mechanisms. Impaired axonal transport **(A)** and interaction with plasma membarne **(B)** can lead to neuronal dysfunction and death. Mutated tau dysfunction as a mitotic spindle MAP may give rise to aneuploidy, while dysfunction as a chaperon protein protecting DNA may result in chromosome aberrations, both different aspects of genome instability **(C)**.

It has been demonstrated that the N-terminal projection domain of tau binds to the p150 subunit of the dynactin complex, which has an essential role in axonal transport, and the attachment of the dynactin to MTs is enhanced by tau (Magnani et al., 2007). R5L and R5H mutations, found in patients with FTLD, affected *in vitro* tau binding to dynactin (Figure 4A). These findings suggest an involvement of tau in axonal transport and imply a subsequent pathogenetic role of tau mutations.

In an *in vitro* system, tau isoforms carrying P301L, ΔN296, and R406W mutations were found to cause a slower kinesin translocation along MT with respect to wt tau (Yu et al., 2014; Figure 4A). As P301L and ΔN296 are localized in the MBD, it was hypothesized that MT assembled and stabilized by mutant tau are structurally and functionally distinct from MT assembled by wt tau, leading to the slowing of kinesin velocities. R406W maps outside the MBD, in the C-terminal region of tau; however, there are evidences that this region influences the MT binding activity of tau (Jeganathan et al., 2006; Mukrasch et al., 2009). These findings provide support to the notion that tau-mediated aberrant modulation of kinesin translocation may lead to neuronal cell death and dementia.

### Tau Interacts with Plasma Membrane

Binding of tau N-terminal projection domain to neuronal plasma membrane had been demonstrated years ago (Brandt et al., 1995). Recent work showed that R406W mutation abolishes tau-membrane binding (Figure 4B), leading to a decreased presence of tau at the tip of neuritis (“loss of function” mechanism; Gauthier-Kemper et al., 2011). Tau-membrane interaction seems to support neurite extension, presumably by bridging the growing MTs to the membrane in the growth cone. This bridging appears to be mediated by annexin A2, an interaction partner of tau. Thus, R406W mutation would compromise tau-annexin A2 interaction, although an influence on tau N-terminal domain cannot be ruled out (Gauthier-Kemper et al., 2011).

Missorting of tau from neurites to somatodendritic compartment is a pathological condition that characterizes neurodegenerative diseases (Zempel and Mandelkow, 2014). R406W mutation thus appears to contribute to this pathological aspect.

### Tau has Nuclear Functions and is Involved in Genome Stability

Tau is localized not only in cytoskeleton, centrosome, and mitotic spindle MT but also in the nucleus (Sjöberg et al., 2006; Rossi et al., 2008a) and has the ability to bind to DNA and protect it from damage in a chaperon-like manner (Hua and He, 2003; Wei et al., 2008). In neuronal cultures, tau can translocate from cytoplasm to nucleus during heath shock, to prevent damage to DNA (Sultan et al., 2011) and a major role for tau in neuronal DNA and RNA protection *in vivo* under physiological and hyperthermic conditions was observed (Violet et al., 2014). In absence of tau, tau knock-out mice exhibited DNA damages such as chromosome missegregation and aneuploidy (Granic et al., 2010).

Tau can be involved in control of chromosome stability based on two different roles (Figure 4C). First, the well-known function as a MAP that regulates spindle MT dynamics. The dysfunction of a MAP can lead to genomic instability (Lengauer et al., 1998) and in fact a mutated tau was demonstrated to produce an unstable and overly dynamic spindle (Alonso et al., 2004; Bunker et al., 2006), which can cause chromosome missegregation and aneuploidy. Second, the interaction of tau with chromatin within the nucleus, that contributes to DNA stabilization and repair. A mutated dysfunctional tau may fail to protect DNA and enhance its damage. In both cases a “loss of function” mechanism can be envisaged.

According to these findings and hypotheses, we found that tau mutations are associated with genome and chromosome instability in peripheral cells of patients affected by FTLD. Cytogenetic analysis of peripheral blood lymphocytes and skin fibroblasts from patients carrying P301L, ΔN296, I10 + 3 G > A; I10 + 16 C > T, G335S, V363I, P364S, G366R, G389R and D418N revealed the presence of several chromosome aberrations such as gaps, breaks, deletions, translocations, aneuploidies, and chromatin anomalies (Rossi et al., 2013). We observed different kinds of aberrations among patients and also between lymphocytes and fibroblasts of the same patient. Thus, mutated tau can be regarded as a predisposing factor to chromosome instability, which appears to be stochastic and involving the whole genome. By in depth analysis of DNA copy number variations through molecular karyotyping, we detected a higher tendency to non-allelic homologous recombination in cells carrying a tau mutation than in cells with wt tau; this suggests a role of mutated tau in this abnormal recombination activity, probably due to its dysfunction as chromatin-associated protein (“gain of function”?; Rossi et al., 2013).

Searching for a confirmation of these results in animal models of genetic tauopathies, we demonstrated that mice expressing P301L and P301S tau mutations have higher aneuploidy levels in their lymphocytes than wt mice (Rossi et al., 2014b). Of course, the cytogenetic study should be extended to CNS, to reveal the presence of chromosome aberrations and to investigate their involvement in neurodegeneration. We hope this investigation will be performed in the future.

## Clinical Features and Diagnostic Implications

Clinical phenotypes of genetic tauopathies are the same as sporadic tauopathies, that is bvFTD, SD, PNFA, PSP and CBS: in fact, tau mutations lead to heterogeneous phenotypes. A high clinical variability is also found both among and within families carrying the same mutation. P301S mutation gave rise to bvFTD in a father and CBD in his son (Bugiani et al., 1999); G389R mutation presented both as bvFTD or CBS (Rossi et al., 2008b); V363I led to bvFTD, SD, PNFA, as well as an atypical phenotype such as posterior cortical atrophy, in some unrelated cases (Rossi et al., 2014a). Thus, to establish a genotype-phenotype correlation within tauopathies is not possible. Moreover, due to clinical overlap between tauopathies and other diseases belonging to FTLD group, which is characterized by genetic heterogeneity, such a correlation is very difficult also in the whole clinical spectrum of FTDL.

When a new tau mutation is found and segregation analysis is not feasible and neuropathological study is not available, a hint about its pathogenicity may come from its position within the protein and comparison to similar previously characterized mutations; however, if possible, an *in vitro* structural and biochemical characterization should be performed to give better indications (Rossi et al., 2012). This would allow to provide affected families with a genetic counseling in which mutations can be attributed at least a likely benign or pathological nature.

A karyotype study on peripheral blood cells may suggest genomic instability; however, as the extent of this phenomenon in CNS in so far unknown, it has not a diagnostic nor prognostic value. We think that the pathological aspect of tau mutations involving DNA damage and chromosome instability should be further investigated for its implication not only in neurodegeneration but also in different diseases associated with genome instability, such as cancer.

## Conclusions

Tau mutations usually alter the primary physiological function of tau as a MAP, that is to promote MT polymerization: in fact, most of missense mutations decrease this ability. This can destabilize the cytoskeleton and its functions in neurons. Mutated tau isoforms show often an increased tendency to self-aggregate into fibrillar deposits and this happens also in the case of mutations causing an imbalance between 3R and 4R tau isoforms. A mass of insoluble, hyperphosphorylated tau thus accumulates within neurons. Cytoskeleton disruption and fibrillar burden are the resulting damages to neuronal cells, leading finally to death.

MAP function of tau can be affected in an opposite way by a small group of mutations that increase the ability of tau to promote MT polymerization. The deriving overstabilization of MT can be detrimental to neuronal function such as axonal transport and synaptic plasticity.

Although fibrillar deposits are usually considered the tau harmful species to neurons, oligomeric tau aggregates have also toxic potential and some rare mutations leading to oligomer formation can explain their pathological effect in this way.

Tau is now more properly considered a multifunctional protein and mutations affecting some roles different from MAP function are emerging: in fact, some mutations were shown to alter dynein and/or kinesin motor proteins activity on MT, being able to slow the axonal flux.

Tau appears also to be involved in genome stability, due to its functions as a MAP and nuclear chromatin-associated protein. Several mutations have in fact been demonstrated to be associated with chromosome structural and numerical aberrations.

Very interestingly, some mutations were investigated for and attributed different pathological features. For example, P301L is a well-known mutation reducing MT polymerization and displaying a great self-aggregation ability; furthermore, it reduced kinesin translocation along MT and was associated with several chromosome aberrations in human and animal cells. Thus, because of the recognized role of tau as a multifunctional protein, a mutation should in turn be considered as potentially having pleiotropic effects disclosing the complex neurodegenerative processes leading to cell death and eventually dementia.

Depending upon the function of tau that is affected and the way it is affected, a tau mutation can cause either a “gain of function” or a “loss of function” condition. A “gain of function” is present in association with fibrillar and/or oligomeric aggregates and in case of MT overstabilization. A “loss of function” possibly occurs in cases of MT destabilization, loss of tau-partner protein interaction, DNA damage and chromosome instability. Complex pathological mechanisms must then be considered when investigating tau-driven neurodegeneration.

Accordingly, therapeutic strategies for tauopathies should take into account all the possibly compromised functions of tau to be successful, targeting tau phosphorylation features and aggregation tendency, oligomer toxicity, MT destabilization/overstabilization (for review, see Boxer et al., 2013; Spillantini and Goedert, 2013; Gerson et al., 2014), as well as DNA and chromosome damage (Rossi et al., 2013, 2014b; Violet et al., 2014), a pathological aspect until now neglected.

## Conflict of Interest Statement

The authors declare that the research was conducted in the absence of any commercial or financial relationships that could be construed as a potential conflict of interest.
